# Insights into the transcriptional regulation of poorly characterized alcohol acetyltransferase-encoding genes (HgAATs) shed light into the production of acetate esters in the wine yeast *Hanseniaspora guilliermondii*

**DOI:** 10.1093/femsyr/foad021

**Published:** 2023-03-25

**Authors:** Isabel Seixas, Diogo Santos, Isabel Vasconcelos, Nuno P Mira, Ana Mendes-Ferreira

**Affiliations:** WM&B—Laboratory of Wine Microbiology & Biotechnology, Department of Biology and Environment, University of Trás-os-Montes and Alto Douro, 5000-801 Vila Real, Portugal; BioISI-Biosystems and Integrative Sciences Institute, Faculdade de Ciências, Universidade de Lisboa Campo Grande, 1749-016 Lisbon, Portugal; iBB, Institute for Bioengineering and Biosciences, Department of Bioengineering, Instituto Superior Técnico, Universidade de Lisboa, Avenida Rovisco Pais, 1049-001 Lisbon, Portugal; Associate Laboratory i4HB—Institute for Health and Bioeconomy at Instituto Superior Técnico, Universidade de Lisboa, Av. Rovisco Pais, 1049-001 Lisboa, Portugal; CBQF/Centro de Biotecnologia e Química Fina, Escola Superior de Biotecnologia, Universidade Católica Portuguesa, 4169-005 Porto, Portugal; iBB, Institute for Bioengineering and Biosciences, Department of Bioengineering, Instituto Superior Técnico, Universidade de Lisboa, Avenida Rovisco Pais, 1049-001 Lisbon, Portugal; Associate Laboratory i4HB—Institute for Health and Bioeconomy at Instituto Superior Técnico, Universidade de Lisboa, Av. Rovisco Pais, 1049-001 Lisboa, Portugal; WM&B—Laboratory of Wine Microbiology & Biotechnology, Department of Biology and Environment, University of Trás-os-Montes and Alto Douro, 5000-801 Vila Real, Portugal; BioISI-Biosystems and Integrative Sciences Institute, Faculdade de Ciências, Universidade de Lisboa Campo Grande, 1749-016 Lisbon, Portugal

**Keywords:** alcohol acetyltransferases, acetate esters and higher alcohols, *Hanseniaspora guilliermondii*, non-saccharomyces yeasts, wine yeasts

## Abstract

*Hanseniaspora guilliermondii* is a well-recognized producer of acetate esters associated with fruity and floral aromas. The molecular mechanisms underneath this production or the environmental factors modulating it remain unknown. Herein, we found that, unlike *Saccharomyces cerevisiae, H. guilliermondii* over-produces acetate esters and higher alcohols at low carbon-to-assimilable nitrogen (C:N) ratios, with the highest titers being obtained in the amino acid-enriched medium YPD. The evidences gathered support a model in which the strict preference of *H. guilliermondii* for amino acids as nitrogen sources results in a channeling of keto-acids obtained after transamination to higher alcohols and acetate esters. This higher production was accompanied by higher expression of the four *HgAAT*s, genes, recently proposed to encode alcohol acetyl transferases. *In silico* analyses of these HgAat’s reveal that they harbor conserved AATs motifs, albeit radical substitutions were identified that might result in different kinetic properties. Close homologues of HgAat2, HgAat3, and HgAat4 were only found in members of *Hanseniaspora* genus and phylogenetic reconstruction shows that these constitute a distinct family of Aat’s. These results advance the exploration of *H. guilliermondii* as a bio-flavoring agent providing important insights to guide future strategies for strain engineering and media manipulation that can enhance production of aromatic volatiles.

## Teaser

Acetate esters production by the wine yeast*Hanseniaspora guilliermondi*.

## Introduction

Acetate esters are a group of aromatic molecules associated to fruity or floral aromas that have been gaining interest as flavorants for application in foods, beverages, fragrances, or cosmetics (reviewed in Saerens et al. 2010, Pires et al. 2014, Dzialo et al. 2017, Holt et al. 2018). Among other interesting properties, acetate esters have a very low odor threshold, which causes them to be detected at very low concentrations and thus to produce detectable sensory alterations even upon minimal alterations in concentration (Saerens et al. 2010). Ethyl acetate, associated to sweet aroma; isoamyl acetate, associated to banana-like aroma; and 2-phenylethylacetate, associated to honey- or rose-like aromas, are among the more interesting flavoring acetate esters, especially in the context of fermented beverages like craft beers or wines (Verstrepen et al. 2003a, Cordente et al. 2012, Dzialo et al. 2017). Along wine fermentation *Saccharomyces cerevisiae* produces isoamyl acetate, 2-phenylethyl acetate, and ethyl acetate (Holt et al. 2018); however, these levels are far below those produced by other Non-*Saccharomyces* species (NSYs) (Ciani and Comitini 2011, Comitini et al. 2011, Jolly et al. 2014, Padilla et al. 2016). Among the higher NSY producers are species of the *Hanseniaspora* genus, and in particular *Hanseniaspora guilliermondii*, whose application has been found to positively impact sensorial profile of wines (Rojas et al. 2001, Moreira et al. 2008, Viana et al. 2008, Medina et al. 2013, Lage et al. 2014, Barbosa et al. 2015) and craft beers (Bourbon-Melo et al. 2021, Matraxia et al. 2021). In this context, the strain explored in this work, *H. guilliermondii* UTAD222 has been used as a co-adjuvant of *S. cerevisiae* for fermentation of different natural musts resulting in wines with enhanced floral aroma due to high contents of 2-phenylethyl acetate (Lage et al. 2014, Barbosa et al. 2015). Despite the many observation studies that have been reporting the success of exploring *H. guilliermondii* to improve production of acetate esters to obtain beverages with distinct aroma profiles, the molecular mechanisms, and the environmental factors governing production of these molecules in this species remains largely uncharacterized.

In *S. cerevisiae*, biosynthesis of acetate esters is mostly achieved by conjugation of an acetyl-CoA molecule to the corresponding higher alcohol, with the acetylation reaction being catalyzed by an alcohol-O-acetyl transferase, also known as AATase. In *S. cerevisiae*, three of such enzymes had been identified: ScAtf1 and ScAtf2, accounting for >50% of the total amount of esters produced (Fujii et al. 1994, Nagasawa et al. 1998, Verstrepen et al. 2003c) and, ScEat1, recently demonstrated as an alternative enzyme involved in synthesis of acetate esters, specially of ethyl acetate (Kruis et al. 2018). Other AATs have been identified in *S. cerevisiae*, like ScEht1 or ScEeb1; however, these have been linked to the production of medium chain fatty acid ethyl esters playing little effect in the synthesis of acetate esters (Saerens et al. 2006). Notably, deletion of all the presumed AATs in *S. cerevisiae* has not abrogated production of acetate esters suggesting the existence of additional enzymes with this activity or different production mechanisms (Kruis et al. 2018). Having in mind, the prior description that *S. cerevisiae* alcohol dehydrogenases have hemiacetal oxidizing activity (Kusano et al. 1998), one possibility is that the formation of acetate esters in this species can also result from dehydrogenation of hemiacetal adducts spontaneously formed between ethanol and acetaldehyde, similarly to what was described in *C. utilis* (Kusano et al. 1999). Reversed esterase activity has also been pointed as source of ester formation in other yeasts (Kallel-Mhiri and Miclo 1993) but so far, no evidences have been obtained that Iah1, the main esterase involved in hydrolysis of acetate esters in *S. cerevisae* (Dank et al. 2018) has such activity. Several fermentation conditions have been found to influence the ability of *S. cerevisiae* to produce acetate esters, mainly by modulating the expression level of *ATF* genes (Lilly et al. 2000, Verstrepen et al. 2003c). In specific it has been observed that *S. cerevisiae* produces higher levels of acetate esters at higher temperatures (Landaud et al. 2001, Molina et al. 2007, Saerens et al. 2008, Birch et al. 2013), at higher glucose levels (Verstrepen et al. 2003b, Saerens et al. 2008) and at higher carbon-to-nitrogen ratios (Piddocke et al. 2009, Dekoninck et al. 2012). On the contrary, production of acetate esters is reduced by aeration and upon supplementation of the growth medium with unsaturated fatty acids (Fujii et al. 1997).

Compared to *S. cerevisiae*, much less is known about the genetics and physiology of the wine yeast *H. guilliermondii* and only recently whole-genome sequences have been disclosed (Seixas et al. 2017, Seixas et al. 2019, Steenwyk et al. 2019), including of UTAD222, the strain explored in this study. Among other traits, the genomic analysis of *H. guilliermondii* UTAD222 resulted in the identification of four genes presumed to be AATs (HGUI_00697, or *HgAAT1*; HGUI_00952, or *HgAAT2*; HGUI_01907, or *HgAAT3*; and HGUI_01910 or *HgAAT4*) and thus likely involved in synthesis of acetate esters (Seixas et al. 2019). However, the similarity of these enzymes with the AATs described in *S. cerevisiae* was very low (around 20% identity) and their function was anticipated based on them harboring motifs usually present in this class of enzymes (Seixas et al. 2019). No robust homologues of ScAATs could also be identified in the genomes of *H. uvarum* or *H. opuntiae*, however, both these yeasts exhibited homologues for the HgAAT genes (Seixas et al. 2019). Recently it has been shown that the deletion of *HuAAT1* reduces formation of ethyl acetate, isoamylacetate, 2-methylbutyl acetate, hexyl acetate, and 2-phenylethyl acetate in *H. uvarum* (Badura et al. 2021), confirming its anticipated function in biosynthesis of acetate esters. The absence of Atf-like proteins in other non-*Saccharomyces* species that produce acetate esters has been reported (Gethins et al. 2015, Löser et al. 2015, Guneser et al. 2022) pointing to the existence of AATs that might not have a primary amino acid sequence identical to canonical Atf’s. In this work, we examined how the production of acetate esters by *H. guilliermondii* is impacted by modifications in relevant environmental parameters such as the carbon-to-assimilable nitrogen ratio (C:N), while also studying how these parameters affect transcriptional regulation of the recently identified *HgAAT* genes and modulate production of acetate esters.

## Materials and methods

### Yeast strains and growth media.

Two yeast strains were used in this study, *H. guilliermondii* UTAD222, isolated from a fermenting grape-juice from the Douro Region (Neto and Mendes-Ferreira 2005) and *S. cerevisiae* UCD522, taken from the Enology Culture Collection, Department of Viticulture and Enology, University of California, Davis, USA. The yeasts were cultivated in yeast extract peptone dextrose (YPD) medium (which contains, per liter, 20 g/L glucose, 10 g/L peptone, 5 g/L yeast extract) or in synthetic grape juice medium (GJM), similar in composition to a typical grape juice (Henschke and V. 1993), with some modifications. In specific, an equimolar mixture of glucose and fructose (200 g/L) was used as the carbon and energy source, while yeast assimilable nitrogen (YAN) was added at an initial concentration of 267 mg YAN/L, supplied as a mixture of di-ammonium phosphate (DAP) and a mixture of aminoacids (40:60), prepared from a stock solution containing per liter: 1 g alanine, 9.1 g arginine⋅HCl, 1.7 g asparagine⋅H_2_O, 3.5 g aspartic acid, 6.22 g glutamic acid⋅HCl, 2 g glutamine, 0.74 g glycine⋅HCl, 2.04 g histidine⋅HCl H_2_O, 2 g isoleucine, 3 g leucine, 3.12 g lysine⋅HCl, 1.5 g phenylalanine, 5 g proline, 4 g serine, 3.5 g threonine, 1 g tryptophan, 0.2 g tyrosine, 2 g valine, and 1.5 g methionine.

### Production of acetate esters along simulated wine fermentations conducted by *H. guilliermondii* or *S. cerevisiae*

Simulated wine fermentations undertaken by *H. guilliermondii* UTAD222 or *S. cerevisiae* UCD522 were performed in GJM medium. For that, starter cultures were prepared by growing the cells overnight in 100-mL flasks, containing 50 mL of GJM with 267 mg YAN/L. The flasks were incubated overnight at 25°C in an orbital shaker set at 150 rpm. On the next day, both strains were inoculated, at a cell density of ∼10^6^ CFU/mL, in 250-mL flasks filled to 2/3 of their volume with GJM. The flasks were fitted with a side-arm port sealed with a rubber septum to allow anaerobic sampling and maintained at 20°C in an orbital shaker set at 120 rpm. Yeast growth was followed by periodic measurement of the optical density (600_nm_) of appropriately diluted culture samples in a Schimadzu UV-2101 spectrophotometer (Schimadzu, Kyoto, Japan) and by determining the number of colony forming units (CFUs) in solid YPD agar plates. Fermentations were monitored daily by weight loss, as an estimate of CO_2_ production, and allowed to proceed until no weight loss was observed. Daily aseptic sampling to determine fermentation parameters and the concentration of different metabolites was performed using a syringe-type system. For different reasons that are explained in the “Results” section, fermentations were also performed in GJM medium having 2% total sugar concentration (named GJM_2%_ and having 10 g/L glucose and 10 g/L fructose) or in GJM supplemented with 664 and 332 mg/L concentration of leucine or of phenylalanine, corresponding to 10-times more than what is present in standard GJM (and thus the media was represented by GJM_Leu10__×_ and GJM_Phe10×_). To maintain the final concentration of 267 mg YAN/L, the amount of DAP added to GJM_Leux10_ or GJM_Phe10×_ was reduced accordingly.

YAN present in the GJM medium along the fermentation was calculated as the sum of primary amino nitrogen (PAN) and ammonium, determined using a Y15 enzymatic autoanalyzer (Biosystems, Barcelona, Spain) and respective appropriate kits supplied by the manufacturer. To determine the amount of 2-phenylethanol, isoamyl acetate, and 2-phenylethyl acetate produced along the different fermentations, a method based on headspace solid-phase microextraction (HS-SPME) was used. Briefly, a Varian 450-GC gas chromatograph (Walnut Creek, CA, USA) coupled to a Varian Saturn 240 MS (Walnut Creek, CA, USA) ion trap mass spectrometer and a Combi-PAL autosampler (Varian Pal Autosampler, Switzerland). In vials of 20 mL, 20 µL of 3-octanol at 50 mg/L was added to 5 mL of sample as the internal standard. The injections were in split mode with a 1:30 ratio and the injector temperature was 220°C. The oven temperature program was 40°C (1 min) to 220°C (30 min) at 2°C/min. The MS detector was operated in Full Scan mode with mass range of 33–350 m/z. All the experiments were run with a divinylbenzene/carboxen/polydimethylsiloxane (DVB/CAR/PDMS) 50/30-mm SPME fiber (Supelco Co., Bellefonte, PA, USA). HS-SPME conditions were 5 min incubation at 45°C, followed by 15 min extraction at the same temperature. 3-methyl-1-butanol and ethyl acetate were analyzed using a Varian CP-3800 GC (Varian, Inc, Walnut Creek, CA, USA) gas chromatograph equipped with a flame ionization detector (GC-FID) and connected to a HP 3396 Integrator. Fifty microliters of 4-methyl-2- pentanol at 10 g/L was added to 5 mL of sample as the internal standard. The sample (1 μL) was injected (split, 1:30) into a CP-WAX 57 CB column (Chrompack) of 50 m × 0.25 mm and 0.2 μm phase thickness. The oven program temperature varied from 40°C (5 min) to 80°C at 3°C/min and from 80°C to 200°C (10 min) at 15°C/min. Injector and detector temperatures were set at 220°C. Carrier gas was H_2_ at 1–2 mL/min.

### Production of acetate esters by *H. guilliermondii* or *S. cerevisiae* in YPD medium

The titers of acetate esters produced by *H. guilliermondii* or *S. cerevisiae* during growth in YPD medium were performed using a methodology similar to the one described above. In particular, starter cultures were prepared by growing the yeast cells overnight in 100-mL flasks, containing 50 mL of GJM with 267 mg YAN/L. The flasks were incubated overnight at 25°C in an orbital shaker set at 150 rpm. Both strains were then inoculated in YPD at a cell count ∼10^6^ CFU/mL. All experiments were conducted in 250-mL flasks filled to 2/3 of their volume fitted with a side-arm port sealed with a rubber septum to allow anaerobic sampling and were maintained at 20°C in an orbital shaker set at 120 rpm. Yeast growth was followed by periodic measurement of the optical density (600 nm) of appropriately diluted culture samples in a Schimadzu UV-2101 spectrophotometer (Schimadzu, Kyoto, Japan) and by counting the CFUs in solid YPD agar plates after incubation at 30°C for 48 h. Aseptic sampling was done daily for assessing growth parameters using a syringe-type system. To determine the amount of 2-phenylethanol, isoamyl acetate, and 2-phenylethyl acetate, the same method based on HS-SPME was used.

### *In silico* analysis of *H. guilliermondii* AAT protein-coding genes and search for orthologues

The coding sequence of the well-characterized *S. cerevisiae* AATs, ScAtf1, and ScAtf2, were aligned with the four predicted AAT enzymes in *H. guilliermondii*, HgAat1, HgAat2, HgAat3, and HgAat4. For that, a ClustalW multiple alignment was undertaken, emphasizing the alterations detected in two motifs that are considered part of active site of these enzymes. To identify orthologues of HgAat’s a best reciprocal hit BLASTP was performed using the nonredundant protein database deposited at NCBI as target. Only proteins whose corresponding alignments with HgAat proteins resulted in pairwise alignments with an *e-*value below *e*^−20^ and an identity above 30% were selected as possible candidate homologues and used for downstream analyses. Phylogenetic tree of HgAats and of described AATs in other yeasts was inferred using MEGA (Kumar et al. 2018) and resorting to the Maximum Likelihood method and JTT matrix-based model.

### Quantification of *HgAAT* gene expression

The expression levels of *HgAAT1, HgAAT2, HgAAT3*, and *HgAAT4* genes during simulated wine fermentations conducted in the different GJM media or in YPD were quantified 12 or 24 h after inoculation. At these selected time points 20-mL aliquots of the cultures were centrifuged (2 205 × *g*, 5 min) and the pellet was immediately frozen in liquid nitrogen and held at −80°C until further use. Subsequent RNA extraction was performed using the classical phenol-chloroform method. Quantification of *HgAAT* gene expression in the different conditions was performed resorting to real time RT-PCR. For this, synthesis of cDNA was performed using the SuperScript III RT kit (Invitrogen) and starting from 2 µg of total RNA. The subsequent PCR amplifications were performed using 100 ng/L of cDNA, 100 nM of each specific primer, and 12 µL of SYBR Green Supermix—UDG with Rox (Invitrogen). All amplifications were carried out in a DNA Engine Opticon instrument (Bio-Rad) with an initial step at 95°C for 5 min followed by 40 cycles of 95°C for 30 s, 60°C for 30 s, and 72°C for 30 s. Samples were analyzed in triplicate. Efficiency of each primer set was evaluated by preparing calibration curves using serial dilutions of cDNA. The gene *HgACT1* was used as an internal control as its expression was found to be stable under the experimental conditions we have used. The sequences of the primers used are listed in Table 1.

**Table 1.** Sequences of primer sets used in this study.

Gene	ORF	Primer	Primer sequence 5′-3′	Amplicon size
*HgAAT1*	HGUI_00 697	HgAAT1–Fwd	ACGGCCACTCTTTCATCAGT	65
		HgAAT1–Rev	TTTATCAGAGCGCATTTCTCT	
*HgAAT2*	HGUI_00 952	HgAAT2–Fwd	ACCTGATGTACAACGAAGGA	194
		HgAAT2–Rev	GTGGCATATTGCCAAAGCCT	
*HgAAT3*	HGUI_01 907	HgAAT3–Fwd	AGATGCCAAGTACTGAAACTGC	198
		HgAAT3–Rev	GATCAATTGGTCATGTGGCT	
*HgAAT4*	HGUI_01 910	HgAAT4–Fwd	AGTTGCAGAAAGACCGTCAA	177
		HgAAT4–Rev	TGAACACTTTGGTGCCACAT	
*HgACT1*	HGUI_00 141	HgACT1–Fwd	GACTCTGGTGACGGTGTTTC	152
		HgACT1–Rev	GCAGTGGTGGTGAAGGAGTA	
*ScATF1*	YOR377W	ScATF1–Fwd	GTACGAGGAGGATTACCA	199
		ScATF1–Rev	GATGATCTCGGTGACAACATCATC	
*ScATF2*	YGR177C	ScATF2–Fwd	AAGCCGTACTACGTTCC	115
		ScATF2–Rev	CGCTCATGTCCATGTTC	
*ScACT1*	YFL039C	ScACT1–Fwd	GATTCTGAGGTTGCTGCTTTGG	142
		ScACT1–Rev	GACCCATACCGACCATGATACC	

## Results

### Contrasting with *S. cerevisiae, H. guilliermondii* production of acetate esters is higher in YPD than in synthetic GJM

**Figure 1.**
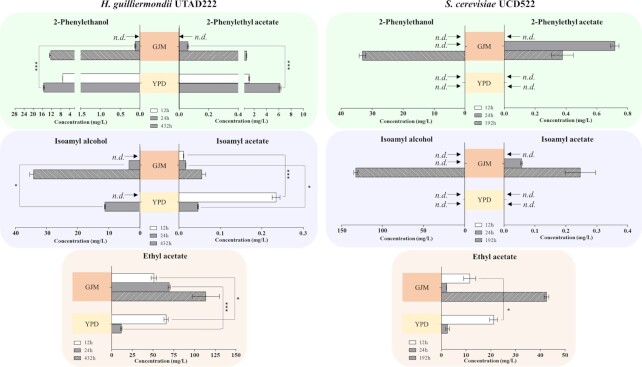
Production of higher alcohols and acetate esters by *H. guilliermondii* is enhanced in YPD (low C: N ratio), while in *S. cerevisiae* it is maximal in GJM (high C:N ratio). After 12 h or 24 h of fermentation undertaken by *H. guilliermondii* UTAD222 or *S. cerevisiae* UCD522 in GJM or YPD the concentration of higher alchols and acetate esters found in the broth was quantified by GC-MS or GC-FID. Samples taken in the end of the fermentation were also obtained (see Supplementary Fig. S2 to see the fermentation profiles obtained by the different yeasts in the two growth media). The results shown are the means of three independent experiments and statistical significance between the results obtained in the different sets of fermentations was assessed using ANOVA. ***P < 0.001, **P < 0.01, *P < 0.05. *n.d*. denotes not detectable.

Previous studies have reported the ability of *H. guilliermondii* UTAD222 to produce acetate esters along fermentations of natural grape-musts, either in single or in mixed culture with *S. cerevisiae* (Lage et al. 2014). However, natural musts are largely variable in terms of their nutritional composition and therefore to obtain more accurate information about the impact of different nutrients in production capabilities of the strain we examined formation of acetate esters along fermentation of a chemically defined synthetic GJM. We focused our attention on the titers of 2-phenylethyl acetate, isoamyl acetate, and ethyl acetate, as well as of their corresponding alcohol precursors 2-phenylethanol and isoamyl alcohol. Production titers were compared during mid-exponential phase (corresponding to 12 h after inoculation, see Supplementary Fig. S1), in the beginning of stationary phase (corresponding to 24 h after inoculation, see Supplementary Fig. S1) and in the end of the fermentation (corresponding to 432 h after inoculation, see Supplementary Fig. S1). To have a standardized point of comparison, production of acetate esters and higher alcohols by *H. guilliermondii* was also followed during cultivation in the rich medium YPD; however, in this case we examined titers only after 12 and 24 h since at this last time-point cells were already achieving stationary phase of growth and sugar was exhausted from the medium (results not shown). During fermentation in GJM medium formation of 2-phenylethyl acetate and 2-phenylethanol prompted by *H. guilliermondii* UTAD222 cells was only detected in the broth after 24 h of inoculation, achieving the highest concentrations in the end of the fermentation, 0.75 mg/L for 2-phenylethylacetate and 12.37 mg/L for 2-phenylethanol (Fig. 1). Notably, the amount of isoamyl acetate achieved in the end of the fermentations was around 10-fold lower the one of 2-phenylethyl acetate, while the differences in concentrations of isoamyl alcohol and 2-phenylethanol was of only 1.4-fold (Fig. 1). This observation suggests that other factors besides the abundance of precursor are limiting the ability of *H. guilliermondii* to produce isoamyl acetate. Compared to the other acetate esters examined, production of ethyl acetate by *H. guilliermondii* in GJM was stronger and faster, reaching a final titer of ∼150 mg/L. Surprisingly, the production of 2-phenylethyl acetate and 2-phenylethanol by *H. guilliermondii* cells in YPD was faster and more robust than the one observed in GJM (Fig. 1). Production of isoamyl acetate in YPD was also faster than in GJM, but strikingly the titers of isoamyl acetate produced during fermentations in YPD declined significantly from 12 to 24 h (∼78% reduction), a trait that was also observed to occur for ethyl acetate (Fig. 1). This reduction is likely to result from degradation of these compounds due to esterase activity. The differences observed in the titers and in the kinetics of acetate esters’ production by *H. guilliermondii* in YPD and in GJM reflect a more rapid sugar degradation (and concomitant higher growth) in the first medium (e.g, at 24 h ∼18 g/L had been consumed from the YPD medium, while in GJM this value was of 10 g/L; Supplementary Fig. S1). However, the differences obtained in the titers of acetate esters goes above 100-fold suggesting that the effect goes beyond a difference in the sugar consumed and in the number of producing cells. Strongly contrasting with *H. guilliermondii*, during growth in YPD *S. cerevisiae* UCD522 cells do not produce detectable amounts of isoamyl acetate, 2-phenylethyl acetate or of the corresponding alcohol precursors, while ethyl acetate was produced (at 12 h) at relatively similar amounts in YPD and in GJM and at much lower levels than those produced by *H. guilliermondii* (for example, production in YPD by *S. cerevisiae* at 12 h was 21.1 mg/L, compared to 65.7 mg/L produced by *H. guilliermondii* in the same time point) (as detailed in Fig. 1).

### Nitrogen and carbon availability modulate the production of acetate esters by *H. guilliermondii*

**Figure 2.**
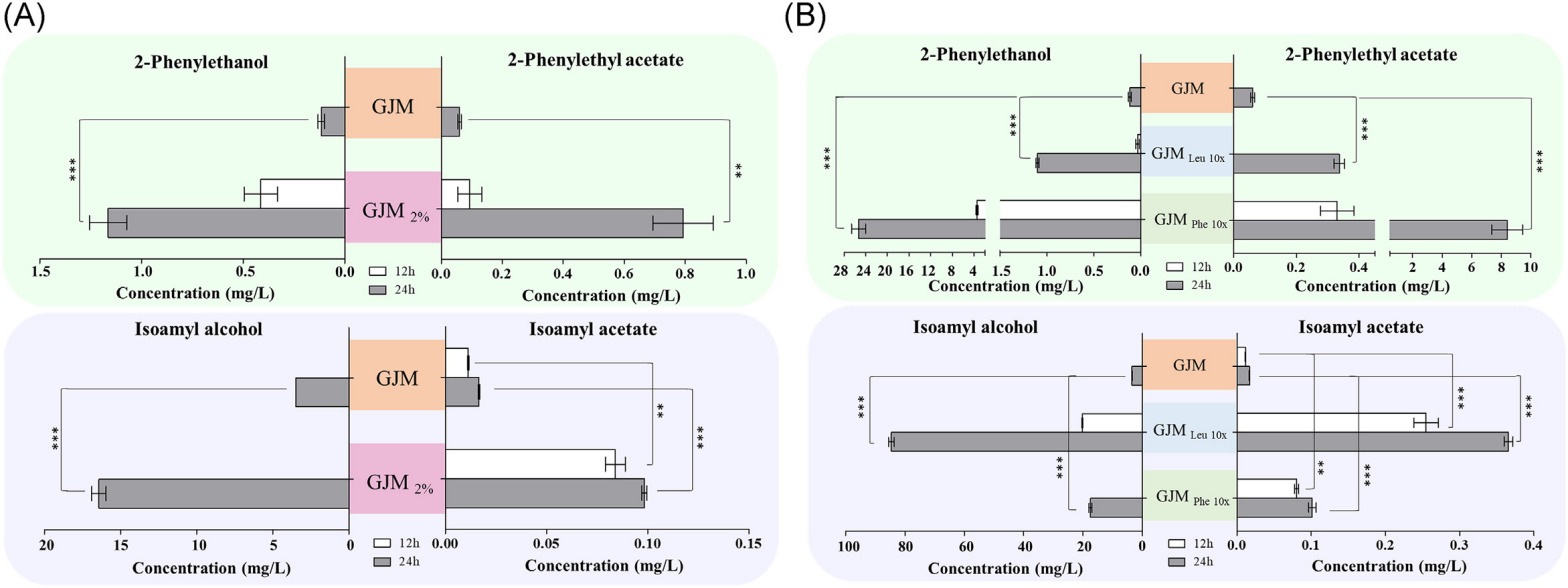
Effect of reducing carbon and increasing concentration of leucine and phenylalanine in the ability of *H. guilliermondii* to produce acetate esters along fermentations in GJM. (A) *H. guilliermondii* UTAD222 cells were inoculated in GJM having 2% total sugars or (B) in GJM medium having a 10× higher concentration of leucine or phenylalanine (assimilable yeast nitrogen was however maintained at the expense of making concomitant reductions in ammonium) and the amount of higher alcohols and acetate esters quantified. In Supplementary Table S2 are provided the values obtained for ethyl acetate that were not significantly different from those obtained in non-supplemented GJM. The results shown are the means of three independent experiments and statistical significance between the results obtained in the different sets of fermentations was assessed using ANOVA. ***P < 0.001, **P < 0.01, *P < 0.05. *n.d*. denotes not detectable.

The very different ability of *H. guilliermondii* cells to produce acetate esters in YPD and in GJM prompted us to take a closer look into the underlying factors, specially because this was also very different from the production capabilities observed in *S. cerevisiae*. Two parameters critical for production of acetate esters differ when we compare the composition of YPD and of GJM: the amount of sugar, much higher in GJM than in YPD (20%, compared with 2%); and the amount of assimilable nitrogen, much higher in YPD than in GJM (736 mg/L YAN, compared to 267 mg/L). Consequently, the carbon-to-assimilable nitrogen ratios in the two media are very different, being 27.2 for YPD (20 g/L sugar, 736 mg/L YAN) and 479 in GJM (200 g/L sugar, 267 mg/L YAN). To assess the influence exerted by sugar concentration in the ability of *H. guilliermondii* to produce acetate esters we repeated the fermentations in GJM but using a final concentration of sugars of 2% (resulting in a GJM_2%_ medium) while maintaining the same amount of nitrogen (267 mg/L). Under these conditions, the ratio of C:assimilable N in GJM_2%_ was of 0.075, contrasting with 0.75 obtained in GJM_20%_. Growth of *H. guilliermondii* UTAD222 cells in GJM_2%_ was above the one observed in GJM_20%_ medium (AUC of 245 compared to 163, Supplementary Fig. S2) and the production of 2-phenylethanol and 2-phenylethyl acetate increased (Fig. 2A). The same trend of higher production in GJM in conditions in which the carbon-to-assimilable nitrogen ratio (C:N) is lower was also observed for isoamyl acetate and isoamyl alcohol, while the levels of ethyl acetate were identical in GJM_20%_ and GJM_2%_ media (Fig. 2A and Supplementary Table S1).

Concerning the effect of nitrogen, this is harder to dissect since the composition of YPD is not defined and therefore it is difficult to estimate the individual contribution of each nitrogen source for the final amount of nitrogen available for the cells. However, YPD has a much higher concentration of PAN (∼700 mg/L, compared to 125 mg/L present in GJM) leading us to hypothesize that the higher production of acetate esters in this medium could result from a higher concentration of amino acids and, in particular, of phenylalanine and/or leucine. To test this idea, we repeated the fermentations in GJM medium with 10× more leucine or phenylalanine (originating media GJM_Leu10×_ or GJM_Phe10×_) while maintaining the final concentration of YAN in 267 mg YAN/L (so we have reduced the amount of ammonium by 77.2% and by 41.2% in GJM_Leu10×_ and GJM_Phe10×_, respectively). The increase in the concentrations of leucine and phenylalanine improved growth of *H. guilliermondii* UTAD222 cells in GJM medium (AUC of 224 for GJM_10×Phe_ and 236 for GJM_10×Leu_, compared to 163 obtained in non-supplemented GJM medium); however, the fermentation profiles remained identical (Supplementary Fig. S3). The increase in precursors resulted in higher concentrations of isoamyl acetate or 2-phenylethylacetate, while the concentration of ethyl acetate remained identical to the one obtained in non-supplemented GJM medium (Fig. 2 panel B and Supplementary Table S1). Interestingly, over supplementation with phenylalanine not only boosted the production of 2-phenylethylacetate by about 107-fold, but also increased production of isoamyl acetate by about 5-fold, compared to the levels obtained in non-supplemented GJM medium (Fig. 2B). Similarly, over supplementation of GJM with leucine resulted in a simultaneous increase of isoamyl acetate and of 2-phenylethyl acetate by 30- and 2- fold, respectively (Fig. 2B). Consistent with these observations, supplementation with either leucine or phenylalanine also increased the titers of both precursors isoamyl alcohol and 2-phenylethanol (Fig. 2B). Altogether this data renders evident that both sugar and amino acids availability impact production of isoamyl acetate and 2-phenylethyl acetate by *H. guilliermondii* cells (while not affecting production of ethyl acetate) being more favorable the utilization of reduced carbon:assimilable nitrogen ratios, unlike what is observed to occur with *S. cerevisiae* whose production of acetate esters occurs at high C:N ratios (Verstrepen et al. 2003b).

### *In silico analysis* of *H. guilliermondii HgAAT*s suggests that these are part of a novel family of alcohol acetyl transferases

**Figure 3.**
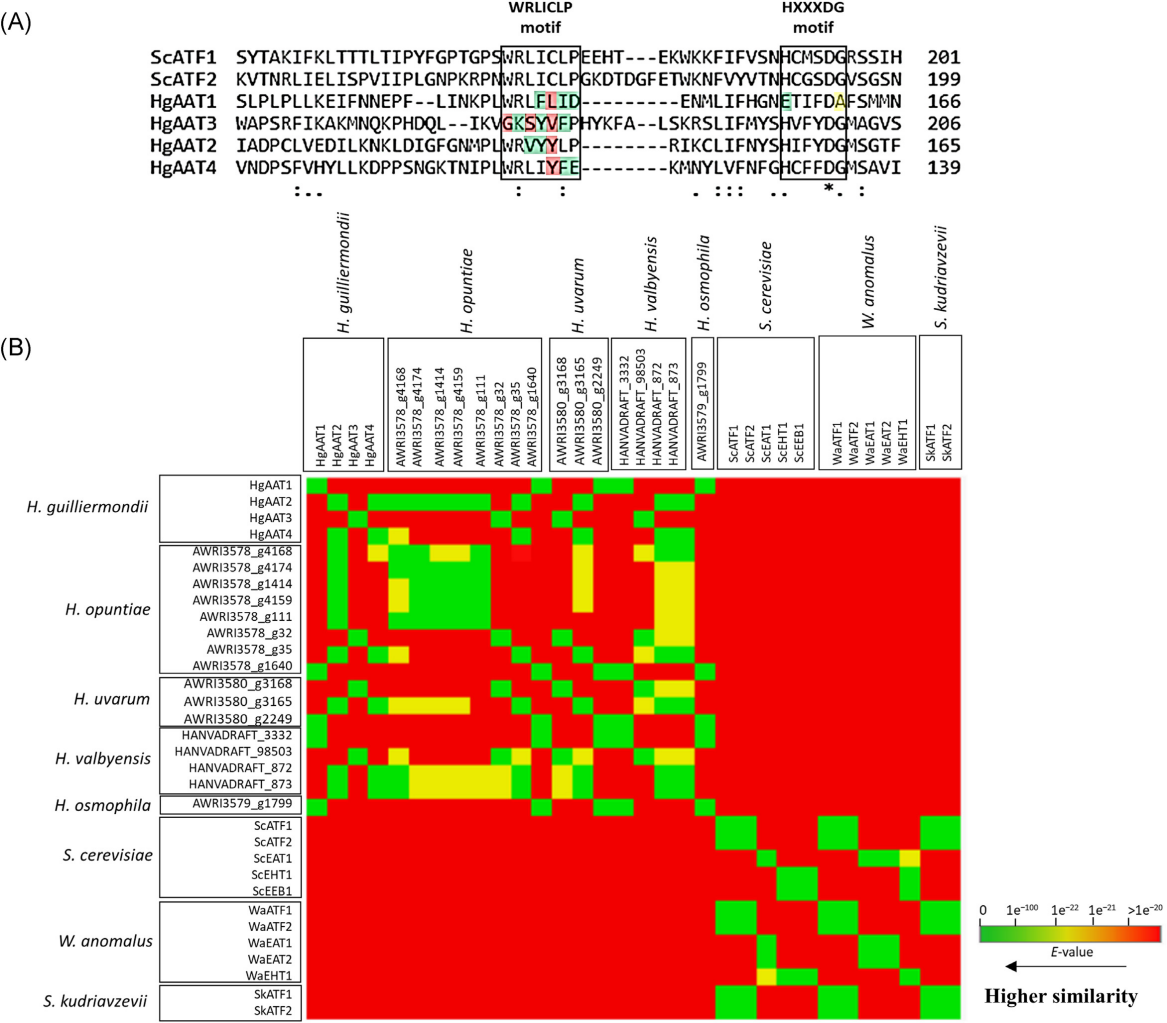
Similarity of the four presumed HgAat’s with close uncharacterized homologues found in species of the *Hanseniaspora* species and with a set of enzymes with described AATase activity in other yeast species. (A) The amino acid sequences of the *S. cerevisiae* ScAtf1, ScAtf2, and of four HgAat’s were aligned using ClustalW (the full alignment can be seen in Supplementary Fig. S1) and the regions spanning the conserved HXXXDG and WRCLIP domains, believed to serve as part of the active site of AATases (see text for references) is shown. Substitutions were classified according with the Grantham score method: substitutions marked in green correspond to conservative or moderately conservative (0 score); substitutions marked in yellow correspond to moderately conservative substitutions (score of 60 or below); and substitutions marked in red correspond to radical or moderately radical substitutions (score above 145); (B) Heat map reflecting the degree of similarity of the four identified HgAat’s with close homologues identified in other species of the *Hanseniaspora* species and with characterized AATs enzyme in *S. cerevisiae, W. anomalus*, and *S. kudriavzvevii*. The heat map denotes the high similarity existing between the four HgAat’s, something that is not observed among AATs from other yeast species that show higher degrees of similarity between themselves. The heat map also shows that homologues of HgAat2, HgAat3, and HgAat4 are only found among the *Hanseniaspora* species *H. uvarum, H. opuntiae, H. valbensysis*, and *H. osmophila*, while HgAat1 is also conserved in *H. osmophila* and in other species from other genera (see text for further details and Supplementary Table S1).

In a previous work, four HgAat predicted enzymes were identified based on them harboring the HXXXDG and WRLICLP motifs (Seixas et al. 2019) hypothesized as part of the active site of AATases (Nagasawa et al. 1998, D’Auria 2006). Herein, the comparative analysis of the amino acid sequence of the different HgAat enzymes with ScAtf1 and ScAtf2 confirm that all these six proteins harbor the HXXXDG motif with the exception of HgAat1 that harbors an EXXXDA motif (Fig. 3A and Supplementary Fig. S4). However, the histidine to glutamate and glycine to alanine substitutions are conservative (according with the Grantham scoring method (Grantham 1974) and thus these alterations are unlikely to affect protein structure. A higher variation was observed within the WRLICLP-motif, with HgAat3 exhibiting six substitutions compared to the canonical motif; HgAat2 and HgAat4 three substitutions each, and HgAat1 four substitutions, as detailed in Fig. 3A. Some of these substitutions are radical (highlighted in red in Fig. 3A) suggesting that they could have an impact in protein structure and, eventually, in activity. Interestingly, the HgAat3 sequence also harbors (between residues 70 and 74) a DXDX(T/V)-motif found in enzymes with phosphatase activity (Collet et al. 1998, Kobor et al. 1999, Sciorra and Morris 2002). To search for possible homologues of the identified HgAAT enzymes we used the corresponding protein sequences to perform a best reciprocal hit BLASTP analysis against the nonredundant database of proteins deposited at NCBI. We could identify 86 robust homologues for these proteins: 66 for HgAat1, 7 for HgAat2, 6 for HgAat3 and 7 for HgAat4; all these found only in genomes of yeasts (as detailed Supplementary Table S2). HgAat2, HgAat3, HgAat4 only exhibited robust homologues in *H. opuntiae, H. uvarum* and *H. valbyensis*, while for HgAat1 we could find homologues in species belonging to the *Lachancea, Torulaspora, Kazachstania*, and *Saccharomyces* genus, besides those found in the *H. guilliermondii*-sister species *H. uvarum* and *H. opuntiae* (Supplementary Table S2 and Fig. 3B). Only one of these identified homologues has a function associated to AATase activity (KAF4002467.1 from *S. cerevisiae* FDAARGOS_613), while the biological function of the others remains uncharacterized (Supplementary Table S2). Compared to the sequence of proteins that have already a well described activity as AATs in other yeast species, it is possible to observe that HgAat proteins (and its closest homologues in *H. uvarum* and *H. opuntiae* species) represent a distinguishable cluster (Supplementary Fig. S5) suggesting that they could represent a novel family of such enzymes. The low similarity exhibited among HgAats was also an observation that stood out considering that this was not observed among other Aat’s already described in other yeast species that bared high degrees of similarity among themselves (Fig. 3B) (Van Laere et al. 2008, Kruis et al. 2018). For example, while the alignment of ScAtf1 with ScAtf2 has an associated e-value of *e^−^^126^*, cross-alignments of HgAat proteins result in alignments with e-values ranging from 0.14 to *e^−^^24^*.

### The higher production of acetate esters is accompanied by higher expression of *HgAAT* genes

**Figure 4.**
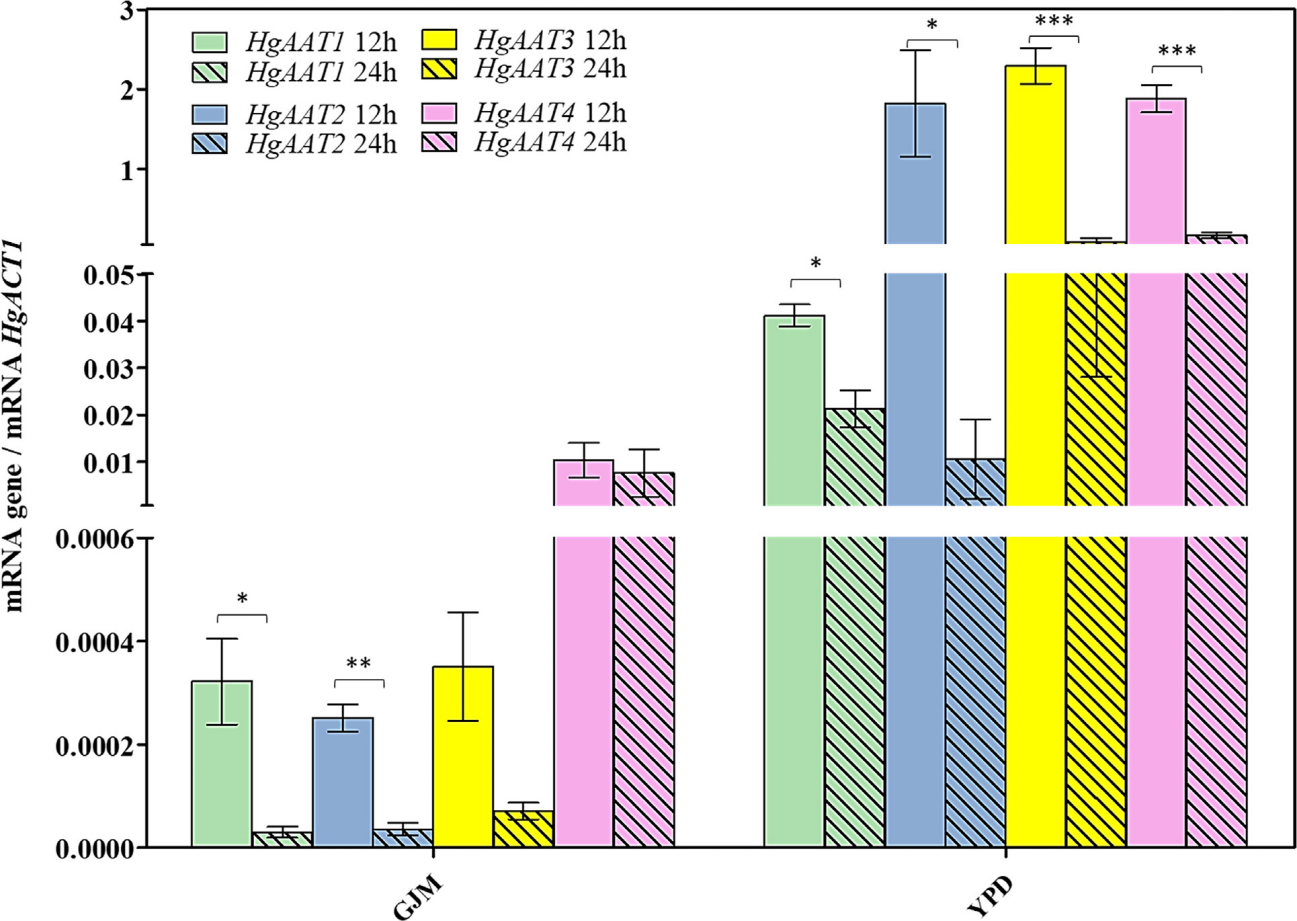
Expression of *HgAAT1, HgAAT2, HgAAT3* and *HgAAT4* genes along fermentations of *H. guilliermondii* UTAD222 in YPD or in GJM. Fermenting cells cultivated in YPD or in GJM were harvested 12 and 24 h prior inoculation (the same time points at which the amount of higher alcohols and acetate esters were quantified) and total RNA extracted as detailed in materials and methods. Gene expression was quantified using real time RT-PCR. *HgACT1* was used as internal control and the gene expression represented corresponds to the ratio between the transcript level of each gene (corresponding to 2^(–Ct)^) and the transcript level obtained for *HgACT1* (corresponding to 2^(–Ct)^) in each sample. The results shown are the means of three independent experiments. Statistical significance between expression at the two time points was assessed using ANOVA, taking into account the different replicate assays performed. ***P < 0.001, **P < 0.01, *P < 0.05.

**Figure 5.**
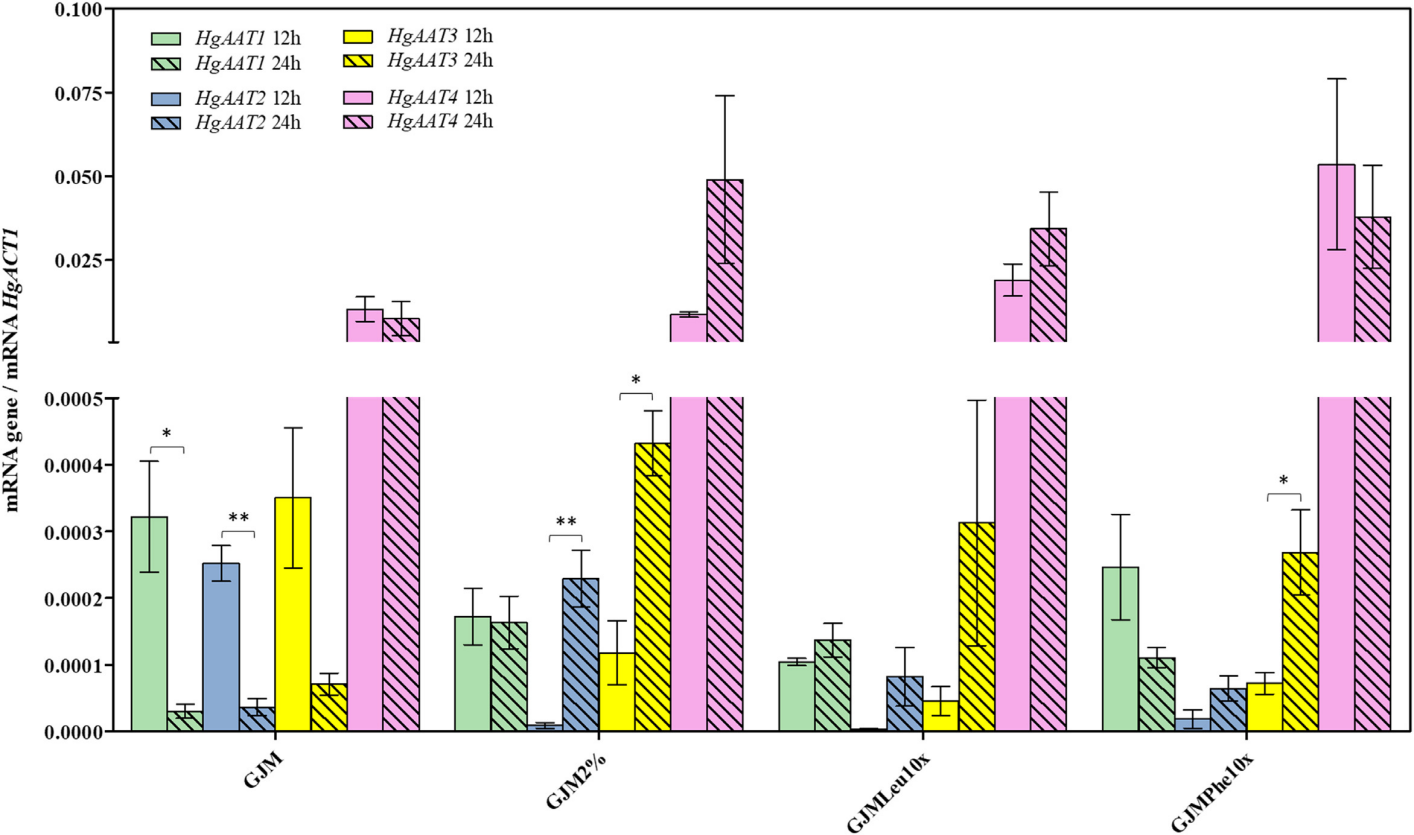
Expression of *HgAAT1, HgAAT2, HgAAT3* and *HgAAT4* genes along fermentations of *H. guilliermondii* UTAD222 in GJM having 2% total sugars (GJM_2%_) or supplemented with leucine (GJM_Leu10x_) or phenylalanine (GJM_Phe10x_). Fermenting cells cultivated in GJM_2%_, GJM_Leu10x_ or GJM_Phe10x_ were harvested 12 and 24 h prior inoculation (the same time points at which the amount of higher alcohols and acetate esters were quantified) and total RNA extracted as detailed in materials and methods. Gene expression was quantified using real time RT-PCR. *HgACT1* was used as internal control and the gene expression represented corresponds to the ratio between the transcript level of each gene (corresponding to 2^(–Ct)^) and the transcript level obtained for *HgACT1* (corresponding to 2^(–Ct)^) in each sample. The results shown are the means of three independent experiments. Statistical significance between expression at the two time points was assessed using ANOVA, taking into account the different replicate assays performed. ***P < 0.001, **P < 0.01, *P < 0.05.

Previous studies demonstrated the correlation between higher expression of ScAtf1/ScAft2 genes and levels of acetate esters produced by *S. cerevisiae* (Yoshimoto et al. 2002, Verstrepen et al. 2003c, Saerens et al. 2008). Having this aspect in mind we examined the expression of *HgAAT1, HgAAT2, HgAAT3*, and *HgAAT4* genes along the fermentations undertaken in GJM or in YPD medium. We monitored the transcript levels of those genes after 12 and 24 h of inoculation in the two media, these also being the time-points at which we have estimated the levels of acetate esters and of the corresponding alcohol precursors. The results obtained demonstrate that all *HgAAT* genes are more expressed in YPD than in GJM, consistent with the higher production of acetate esters by *H. guilliermondii* cells in the first medium (Fig. 4). This difference in expression was particularly evident for *HgAAT2, HgAAT3*, and *HgAAT1*, whose difference in transcript levels reached 6000-, 5725-, and 190-fold (Fig. 4). Along cultivation in GJM *HgAAT4* was much more expressed than the other *HgAAT* genes (Fig. 4) and maintained at a stable transcript level between 12 and 24 h of cultivation, while expression of *HgAAT1, HgAAT2*, and *HgAAT3* genes decreased prominently in this time interval (Fig. 4). In YPD these same trends were observed although the expression differences between 12 h and 24 h were smaller, likely due to an already higher basal expression of all the *HgAAT* genes (Fig. 4). We have also monitored the expression of *HgAAT* genes along the fermentations undertaken in GJM_2%_, GJM_Phe10x_ and GJM_Leu10x_. The most prominent difference prompted by the reduction in GJM_2%_ medium was the increased expression of *HgAAT1, HgAAT2* and *HgAAT3* at 24 h of these genes, comparing with those attained in GJM_20%_ (Fig. 5). The increased expression of these *HgAAT* genes in GJM_2%_ was noteworthy as it correlates with the higher production of 2-phenylethyl acetate and isoamyl acetate (Fig. 5). The increase in the availability of leucine and phenylalanine (and the concomitant decrease in ammonium) reduced the expression at 12 h of *HgAAT1, HgAAT2* and *HgAAT3* genes; but at 24 h the expression levels of these three genes was already similar or even above (e.g. *HgAAT3*) the one observed in cells cultivated in non-supplemented GJM medium (Fig. 5). It is important to stress that in the GJM_Phe10x_ and in the GJM_Leu10x_ there was a concomitant reduction in the amount of ammonium phosphate provided in the medium and, therefore, we can not rule out a possible alleviation of an eventual repressive effect exerted by this inorganic nitrogen source over expression of *HgAAT* genes

## Discussion

Currently there is an increased interest in the exploration of Non-*Saccharomyces* yeasts and, in particular, of *Hanseniaspora* species as possible bio-flavouring agents due to their increased ability to produce acetate esters (Rojas et al. 2001, Viana et al. 2008, Testa et al. 2020). Such exploration is, however, hampered by the lack of knowledge on the molecular mechanisms that underlie these production capabilities, including the specific identification of the catalytic enzymes involved, as well as environmental factors that modulate production. Although a lot of knowledge has been gathered concerning these aspects in the budding yeast *S. cerevisiae* (as reviewed in Holt et al. 2018), genetics and physiology of this species varies significantly from what is observed in NSYs and therefore extrapolation is limited, if any. The results presented in this work confirm precisely that, with acetate esters production capabilities of *H. guilliermondii* significantly varying from those observed in *S. cerevisiae*, as discussed below.

**Figure 6.**
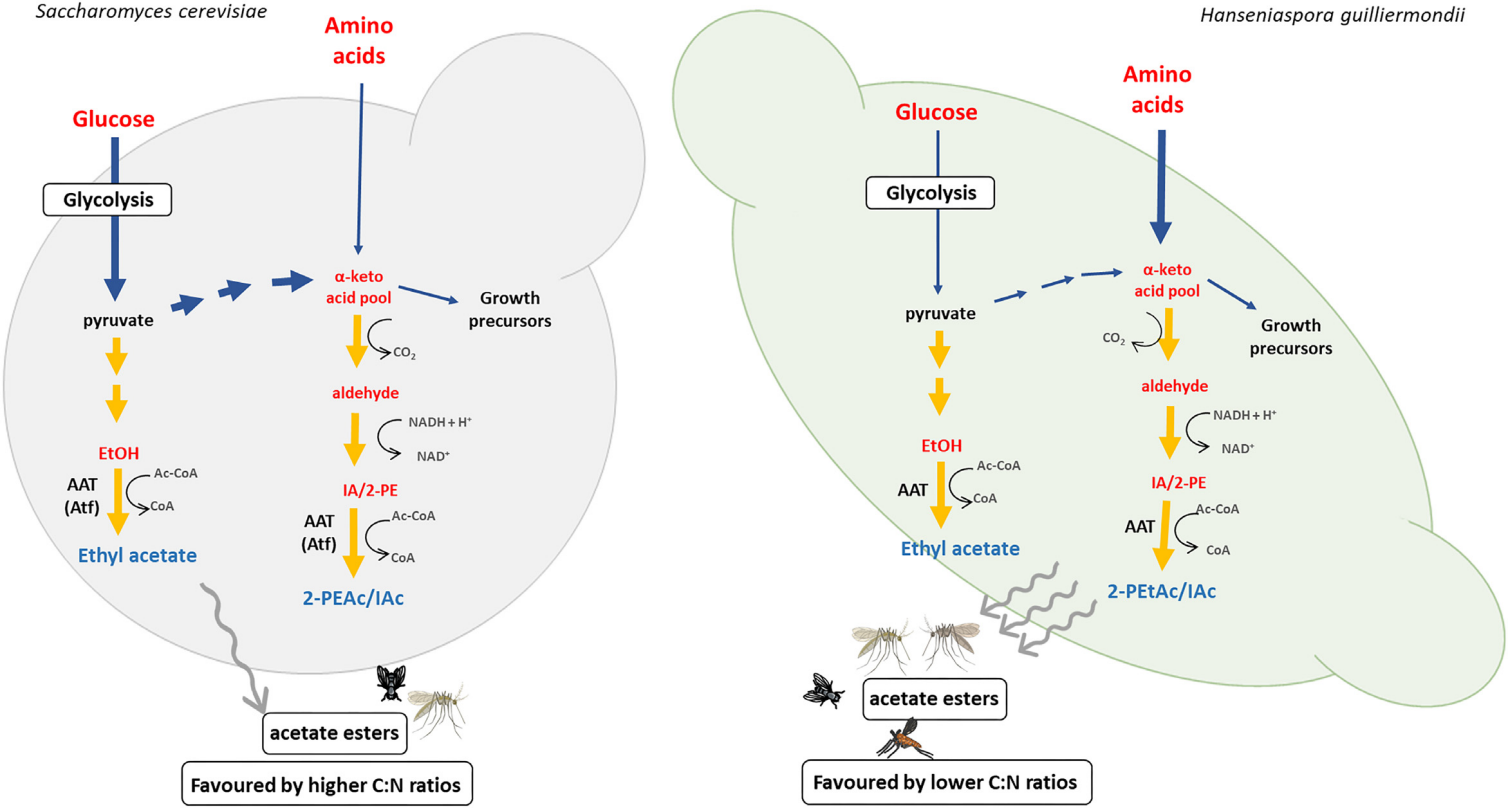
Schematic representation of acetate ester production in *S. cerevisiae* and in *H. guilliermondii*. While in *S. cerevisiae* production of acetate esters is driven by keto-acids produced by the Krebs cycle (see text for references) in a growth-dependent manner, in *H. guilliermondii* the precursors are the amino acids assimilated from the growth medium. The data obtained in this study lead us to propose that HgAat4 is likely involved in synthesis of ethyl acetate, while no specific assignment could be made for HgAat1, HgAat2 and HgAat3. IA: isoamylalcohol; 2-PE: 2-phenylethanol; 2-PEtAc: 2-phenylethyl acetate; IAc—isoamyl acetate.

It is herein demonstrated that *H. guilliermondii* cells over-produce acetate esters when the ratio of C: assimilable N is reduced, strongly contrasting with *S. cerevisiae* that produces these volatiles at high C:N ratios in a growth-coupled manner (Verstrepen et al. 2003b, Saerens et al. 2008). The higher production titers at lower C:assimilable N ratios is not specific of our UTAD222 strain since a similar result had also been obtained in another study that used a different strain of *H. guilliermondii* (Viana et al. 2008). Also, the synthesis of aromatic alcohols by *M. pulcherrima* was also significantly higher in low glucose (2%) media (González et al. 2018). Using radioisotope tracing it was demonstrated that the precursors for synthesis of volatile aroma compounds by *S. cerevisiae* along wine fermentation are keto-acids mainly obtained from central carbon metabolism, with the contribution of amino acid catabolism being minor (Crépin et al. 2017, Rollero et al. 2017). Such metabolic routing is consistent with the dependence of growth for production of acetate esters by *S. cerevisiae*. Accumulation of acetate esters in fermentation broth carried out by *H. guilliermondii* is only detectable when cultures achieve stationary phase indicating that production is not directly coupled to growth. We propose that these differences to *S. cerevisiae* result from *H. guilliermondii* having a preference for amino acids as nitrogen sources that, upon assimilation, are channelled for production of higher alcohols and acetate esters. This hypothesis is supported by several lines of evidence: (i) *H. guilliermondii* cells do not consume any of the ammonium provided in GJM or in YPD, while the amount of amino acids is prominently decreased in all the fermentations (see Supplementary Fig. S6); (ii) the genome of *H. guilliermondii* encodes only one ammonium permease (but 10 putative general amino acid permeases) and these cells are devoid of the NADP^+^-ammonium fixation enzymes Gdh1 and Gdh3 (Seixas et al. 2019), resulting in a higher difficulty to fixate ammonium; (iii) growth of *H. guilliermondii* cells is higher in the leucine- and phenylalanine GJM medium, compared to non-supplemented GJM (and here it is important to stress that GJM_Leux10_, GJM_Phe10x_, and GJM all have 267 mg/L of YAN since the increase in leucine or phenylalanine was compensated with a decrease in ammonium phosphate levels); *iv)* production of acetate esters by *H. guilliermondii* is enhanced in amino acid-enriched media (such as YPD or GJM supplemented with leucine and phenylalanine). Thus, while *S. cerevisiae* uses the production of acetate esters to avoid unwanted/unnecessary accumulation of keto-acids derived from the Krebs cycle along sugar catabolism, *H. guilliermondii* cells synthesizes acetate esters as a mean to retain NH_2_ group ‘getting rid of the unwanted/unnecessary carbon’ (coming from the assimilated amino acids) in the form of volatile higher alcohols/acetate esters (as schematically represented in Fig. 6). Other advantages from this approach are the enhanced recycling of NADH prompted by the synthesis of higher alcohols and the antimicrobial potential of higher alcohols (Liu et al. 2014) that can reduce the activity of competing microbes in the environment.

Detailed comparative analyses of the putative *HgAAT*s identified while mining *H. guilliermondii* genome confirmed prominent differences between these proteins and other already described AATs, including modifications (more visible in HgAat3) in the highly conserved motifs believed to serve as active sites for these enzymes. HgAat2, HgAat3, and HgAat4 appear to constitute a sub-group of AATs exclusive of the closely related species *H. guilliermondii, H. uvarum, H. opuntiae* (*H. valbyensis* also exhibits homologues but these are less similar to those found in the other three *Hanseniaspora* species), while HgAat1 is closer to other described AATs. One possibility behind the distinguishing acetate esters producing ability of *H. guilliermondii* is the existence of this specific set of enzymes that may have better catalytic properties [e.g. higher affinity for the substrate(s)], compared to their counter-partners found in other species. The catalytic properties of Atf enzymes among species of the *Saccharomyces sensu strictu* were found to vary considerably, with enzymes from *S. uvarum* and *S. eubayanus* exhibiting affinity towards substrates 2- to 3-fold higher than those found in the corresponding *S. cerevisiae* orthologues, resulting in enhanced acetate esters production (Stribny et al. 2016). In particular, Atf enzymes from *S. uvarum* and *S. kudriavzevii* were found to display a broader range of substrate specificity, compared to *S. cerevisiae*, resulting in enhanced capacity to over-produce isoamyl acetate and ethyl acetate, but also 2-phenylethylacetate, structurally different (Stribny et al. 2016). Similarly, HgAats can also have broader substrate ranges considering the recognized capabilities of *H. guilliermondii* to produce 2-phenylethyl acetate and ethyl acetate (also evidenced by the results herein obtained), two traits for which this species is long recognized for (Rojas et al. 2001, Rojas et al. 2003, Moreira et al. 2005, Lage et al. 2014). Detailed biochemical studies assessing the kinetic properties of HgAat’s will be required to answer these open questions, also addressing the impact of the modifications found in the conserved active sites. Notably, 2-phenylethyl acetate, isoamyl acetate, and ethyl acetate were found to serve as potent attractors for insects (Schubert et al. 2014, Giang et al. 2017) forming a potent vehicle for dissemination of yeasts in vineyards (Fogleman et al. 1982, Piper et al. 2017, Stefanini 2018). *H. guilliermondii, H. uvarum*, and *H. opuntiae* are indigenous to wine musts and it is possible that their enhanced ability to produce acetate esters potentiates their dissemination in this environment. The identification of *H. guilliermondii* and *H. uvarum* in the gut microbiome of fruit flies (Lam and Howell 2015, Piper et al. 2017) substantiates further the occurrence of this yeast-insect interaction, potentially enhanced by their high ability to produce acetate esters.

Higher production of acetate esters by *H. guilliermondii* was accompanied by higher expression of *HgAAT* genes, supporting the anticipated involvement of these enzymes in this process. In specific, the much higher production of acetate esters by *H. guilliermondii* in YPD (having a low C:N ratio) was accompanied by an increase in transcripts of *HgAAT* genes that reached >1000-fold for *HgAAT2* and *HgAAT3*, compared to the levels attained in GJM (having a high C:N). *HgAAT4* was the gene more actively transcribed in all conditions and also the one whose expression suffered less changes in the different fermentation media used. This observation leads us to suggest that this enzyme is likely responsible for synthesis of ethyl acetate since production of this volatile was considerably higher than 2-phenylethylacetate or isoamylacetate; and production of ethyl acetate was marginally affected by the changes made in the fermentation media. In support of this idea, *HuATF1* was recently implicated in production of ethyl acetate by *H. uvarum* (Badura et al. 2021), this enzyme sharing with HgAat4 the same exact WRLIYF-motif in the presumed active site. With the data available we cannot assign HgAat1, HgAat2 or HgAat3 to the specific production of ethyl acetate, 2-phenyl ethyl acetate or isoamyl acetate; however, this is an essential next step to guide the engineering of *H. guilliermondii* strains with directed capabilities to produce one of the esters in detriment of the other(s). The accumulation of higher concentrations of 2-phenylethanol and isoamyl alcohol, compared to the corresponding acetate esters, in all the conditions examined (2-phenylethanol is about 2–3-fold more abundant than 2-phenylethylacetate and isoamyl alcohol is 20- to 300-fold more abundant than isoamyl acetate) indicates that the availability of the corresponding AAT enzyme(s) is a limiting factor for production of the acetate ester, as also demonstrated to be the case in *S. cerevisiae* (Verstrepen et al. 2003c, Holt et al. 2018). The pool of available cytosolic acetyl-CoA is likely to be an conditioning factor as well. In fact, *Hanseniaspora* species are known as important acetic acid producers, including in conditions in which over-production of acetate esters has also been observed, although important inter-species and inter-strain variability has been observed. Notably, *H. guilliermondii* strains, and the UTAD222 strain in particular, are among the lower producers (Lage et al. 2014, Barbosa et al. 2015). It is possible that *Hanseniaspora* species, and *H. guilliermondii* in particular, can have a metabolic network that favors a higher internal pool of acetate enhancing supply of acetyl-CoA for the synthesis of acetate esters, compared to other yeast species. Further studies addressing detailed analyses of *Hanseniaspora* metabolome will shed light into this aspect. The availability of phenylalanine and leucine, direct precursors of acetate esters, was also herein found to influence production; however, the effect was beyond the obvious since supplementation with leucine stimulated production of 2-phenylethanol/2-phenylethyl acetate and phenylalanine stimulated production of isoamyl alcohol/isoamyl acetate. This crossed-stimulation is in line with a higher capacity of *H. guilliermondii* to use the overall available pool of amino acids present in the leucine- and in the phenylalanine- supplemented GJM medium (see Supplementary Fig. S6). What these results suggest is that *H. guilliermondii* cells are able to sense and respond to variations in the individual balance of the different nitrogen sources that are present in its environment (again remember that the GJM_Leu10x_, GJM_Phe10x_, and GJM all have the same YAN), with consequences for the production of higher alcohols and acetate esters and of other metabolites stemming for amino acid catabolism. We can not also rule out that this higher production may result from an alleviation of an eventual repressive effect exerted by ammonium (since the increase in Phe and Leu was accompanied by a decrease in the amount of ammonium phosphate to maintain the overall YAN). Further studies will be required to elucidate this aspect, specially focusing how nitrogen regulation happens in *H. guilliermondii*, although the insights suggested from this work appear to point to prominent differences toward what is described in *S. cerevisiae* and also in *H. vinae*, that has a similar NCR response (Lleixà et al. 2019).

The much more prominent expression of *HgAAT1, HgAAT2*, and *HgAAT3* along *H. guilliermondii* fermentation of amino acid-enriched YPD, contrasts with the very low expression of *ScATF1* and Sc*ATF2* during *S. cerevisiae* UCD522 fermentations in the same conditions (Supplementary Fig. S7) and correlates with the acetate esters titers produced by the two species. In *S. cerevisiae*, the transcriptional regulatory network governing the expression of *ATF* genes in the context of wine fermentation has not been fully clarified but a model has been proposed (Holt et al. 2018). According to this model, the C:N ratio affects the expression of *ATF* genes via Rap1, a central regulator of growth in *S. cerevisiae* that can serve both as activator and a repressor of target gene expression, depending on its interacting partners. Thus, Rap1 would activate *ATF* genes at high C:N ratios due to an interaction with Gcr1, responsive to glycolytic flow; and would repress *ATF* genes at low C:N ratios due to an interaction with Gcn4, a key regulator of nitrogen catabolite repression (NCR) system (Holt et al. 2018). NCR is a complex and intertwined regulatory system that allows *S. cerevisiae* to respond precisely to fluctuations in the nitrogen availability assuring the ability of the cells to favor catabolism of preferred nitrogen sources (with ammonium being one of them)(Gobert et al. 2019). The genome of *H. guilliermondii* does not encode homologues for ScRap1, ScGcn4, or ScGcr1 and we could also not identify homologues of the other *S. cerevisiae* transcription factors involved in NCR (Seixas et al. 2019). This does not necessarily mean that proteins with these functions are absent in *H. guilliermondii* since transcription factors evolve rapidly and functional conservation may occur without conservation of the primary amino acid sequence. However, the herein demonstrated inability of *H. guilliermondii* to use ammonium in the presence of amino acids clearly indicates that the underlying transcriptional system governing response to nitrogen availability is very different from the one active in *S. cerevisiae*. Future studies will have to be performed to understand responses to nitrogen availability in this species and whether these regulators mediate control of *HgAAT* gene transcription and, consequently, regulate production of acetate esters. *S. cerevisiae* strains with enhanced capacities to produce acetate esters had been obtained upon engineering of systems involved in regulation and signaling of nitrogen metabolism (Holt et al. 2018) indicating that this could also be an interesting strategy to obtain over-producing *H. guilliermondii* strains. The release of UTAD222 genomic sequence, as well as of other *H. guilliermondii* strains, is expected to further accelerate these functional studies focused not only on genes causative of the high acetate esters production phenotype, but also a better understanding of how environmental parameters modulate physiology of this yeast and, thus, affect production. Such combined knowledge is essential to guide bioprocesses in which *H. guilliermondii* can be successfully explored as a cell factory, as it is the case of acetate esters production. This work certainly provides the first steps into this matter having demonstrated, for the first time, the effect of C:assimilable N ratio in modulating production, while also clearly implicating *HgAAT* genes as underneath enzymes.

## Supplementary Material

foad021_Supplemental_Files
