# Supplementary material for: Insights into the transcriptional regulation of poorly characterized alcohol acetyltransferase-encoding genes (HgAATs) shed light into the production of acetate esters in the wine yeast Hanseniaspora guilliermondii
Source: FEMS Yeast Res. 2023 Mar 25;23:foad021. doi: 10.1093/femsyr/foad021 (PMC10070066; doi:10.1093/femsyr/foad021)
Supplement: foad021_Supplemental_Files [file foad021_supplemental_files.zip › Supplementary_figures.pdf]

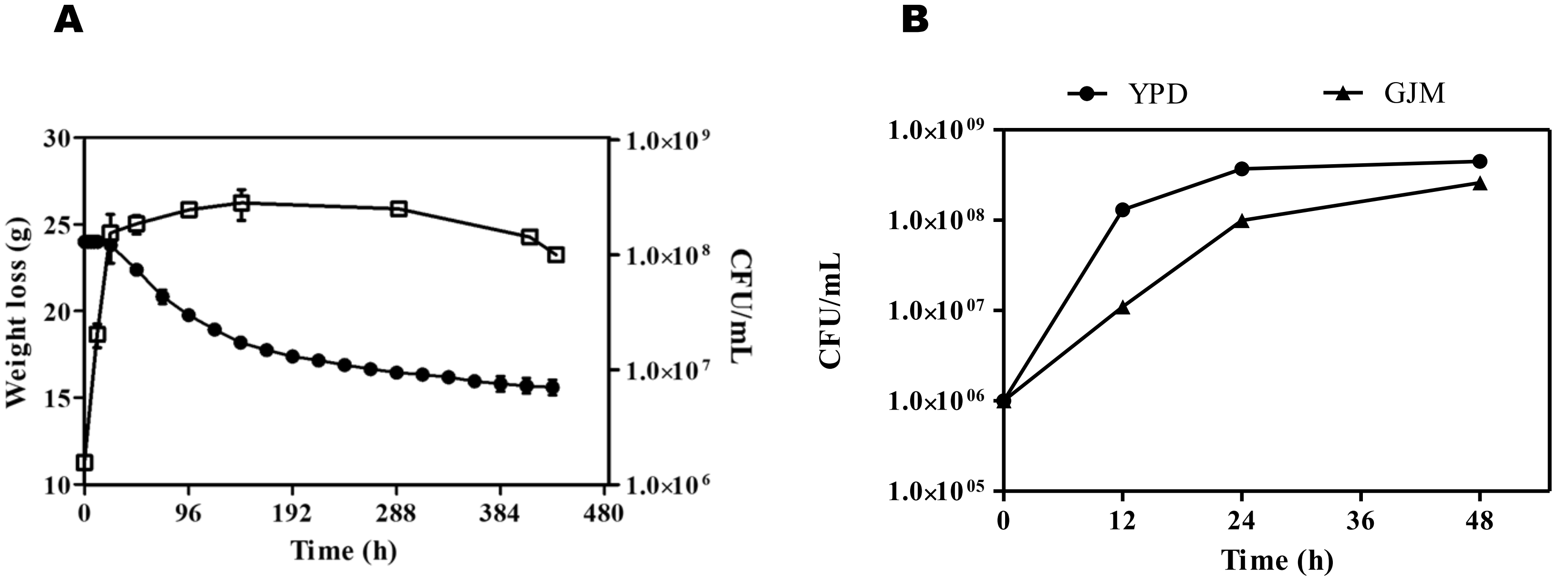

Supplementary Figure S1. (A) *H. guilliermondii* UTAD222 fermentation kinetics and growth profile in GJM. Filled circles refer to weight loss (g) as an estimate of CO_2 production. Empty squares refer to the viable yeast cell number (CFU/mL) of *H. guilliermondii* along the fermentation. (B) Growth curve, estimated based on OD_{600nm}, of *H. guilliermondii* cells along cultivation in YPD. For the sake of comparison the result obtained in the first 48h of fermentation in GJM are also shown. Error bars represent standard deviation obtained for the three replica experiments performed.

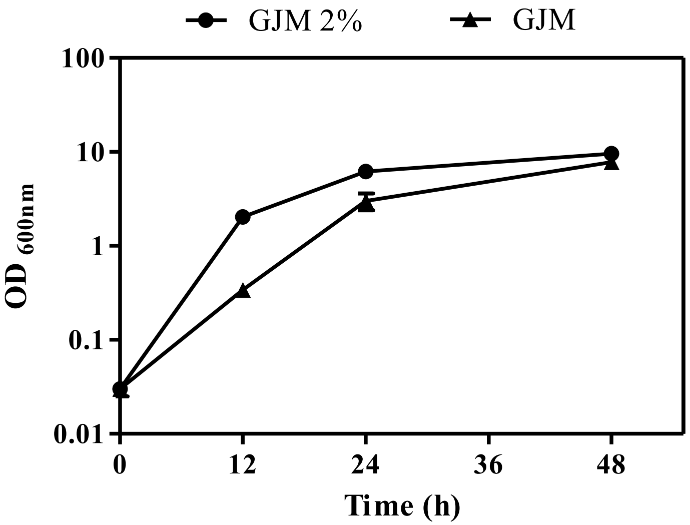

	Growth parameters			
	OD 600 nm			AUC
	12h	24h	48h	48h
**GJM**	0.34 ± 0.03	3.67 ± 0.61	7.82 ± 0.14	163.33 ± 9.67
**$GJM_{2\%}$**	2.04 ± 0.05	6.18 ± 0.09	9.56 ± 0.04	244.67 ± 1.25

Supplementary Figure S2. Growth curves, estimated based on OD600nm, of *H. guilliermondii* cells along cultivation in GJM or in $GJM_{2\%}$. For the sake of comparison the result obtained in the first 48h of fermentation in GJM are also shown. Error bars represent standard deviation obtained for the three replica experiments performed.

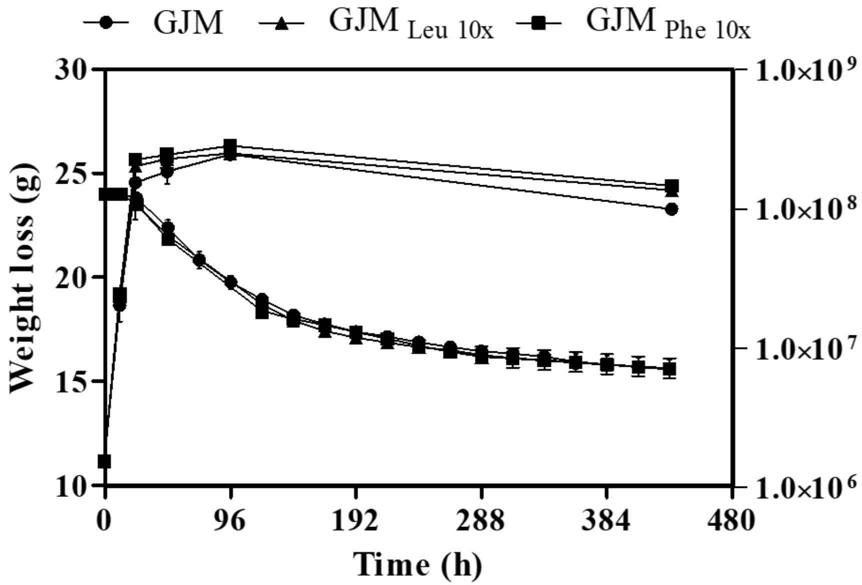

	OD $_{600\ nm}$			AUC
	**12h**	**24h**	**48h**	**48h**
**GJM**	0.34 ± 0.03	3.67 ± 0.61	7.82 ± 0.14	163.33 ± 9.67
**GJM $_{Leu10x}$**	0.39 ± 0.01	4.68 ± 0.20	12.33 ± 0.38	236.00 ± 7.48
**GJM $_{Phe10x}$**	0.39 ± 0.01	4.67 ± 0.16	11.35 ± 0.33	224.00 ± 6.16

Supplementary Figure S3. Growth and fermentation profiles of *H. guilliermondii* in GJM_{Leu10x} and GJM_{Phe10x} compared to GJM. Weight loss (g) was used as an estimate of CO_2 production.

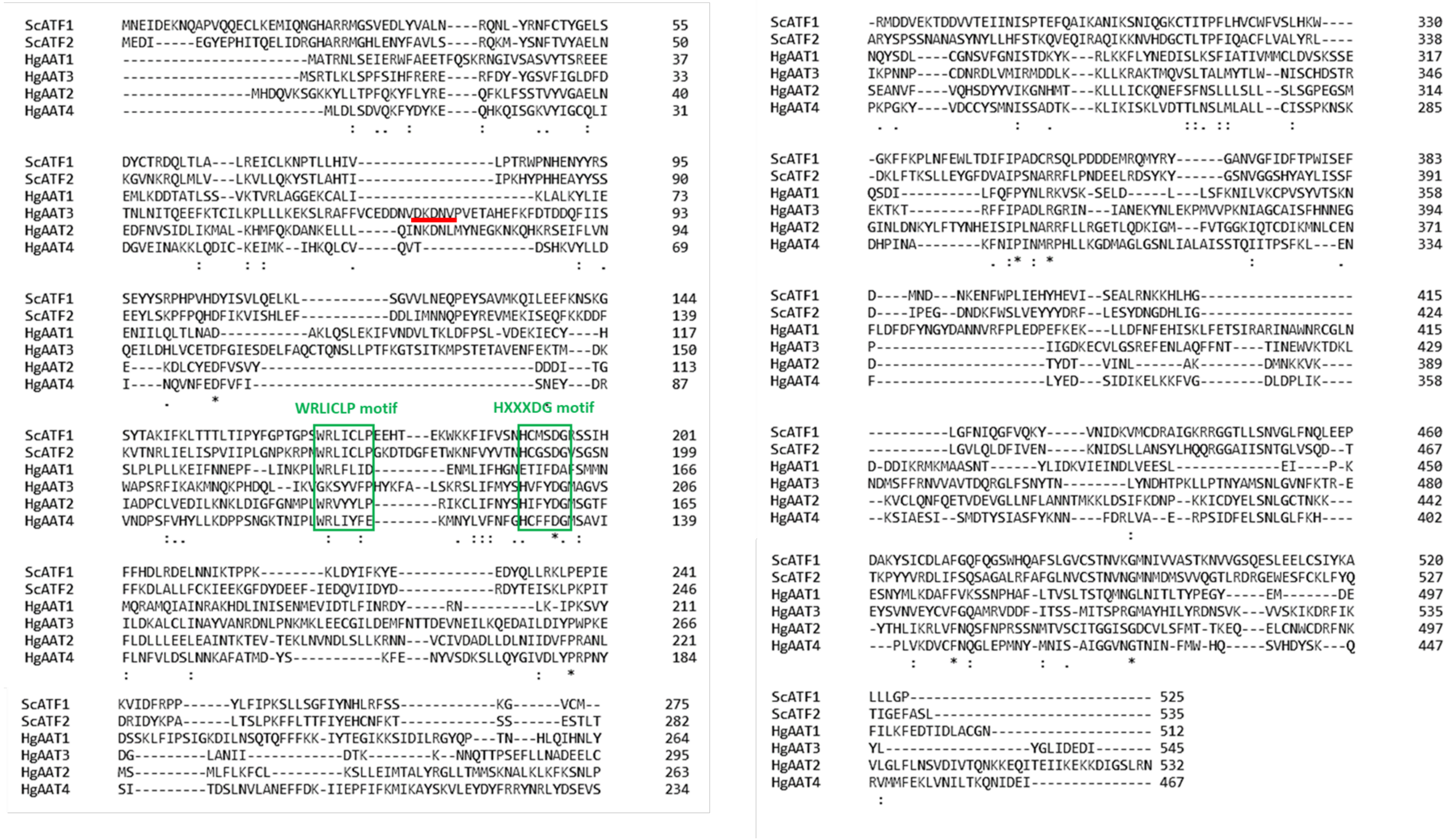

Supplementary Figure S4. Complete alignment of the amino acid sequences of the identified HgAATs and of *S. cerevisiae* Atf1 and Atf2 enzymes. Motifs WRLICLP and HXXXDG, conserved in the active site of AATases, are shown in boxes.

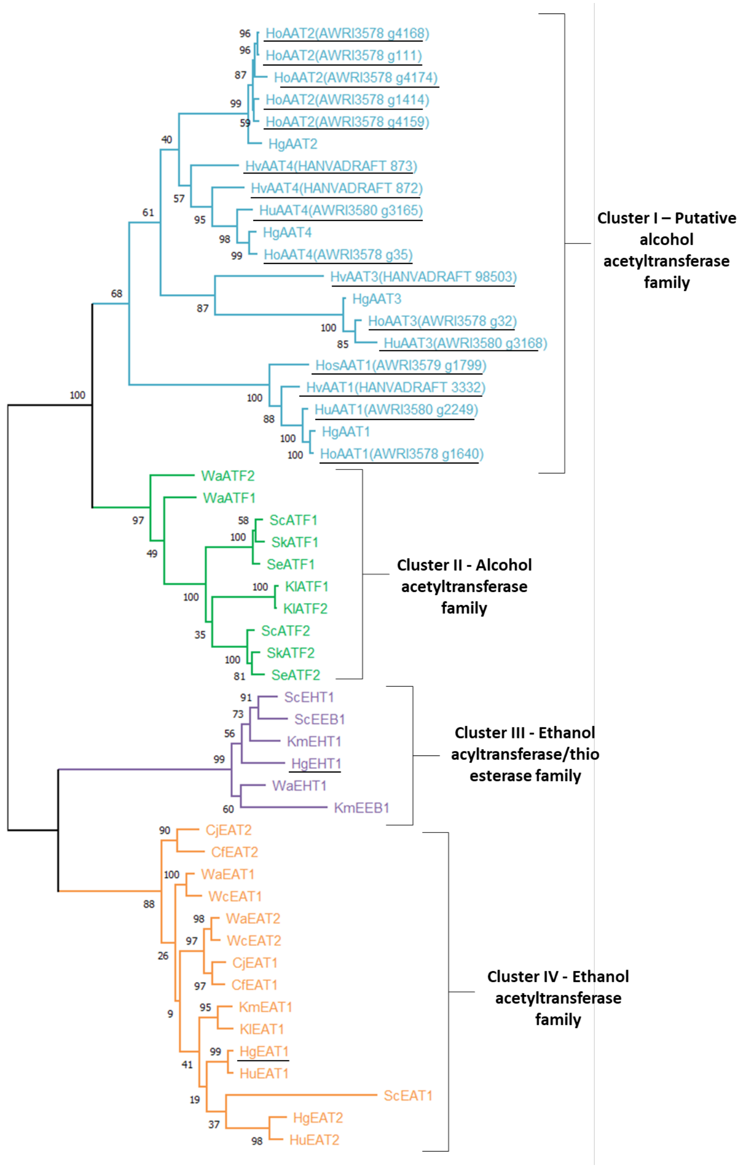

Supplementary Figure S5. Phylogenetic tree describing the positioning, in terms of similarity, of the HgAat's in comparison with its close homologues found in other *Hanseniaspora* species and with a set of described AATases in other yeast species: Cj, *Cyberlindnera jadini*i; Cf, *C. fabianii*; Hg, *H. guilliermondii*; Ho, *H. opuntiae*; Hu, *H. uvarum*; Hv, *H. valbyensis*; Hos, *H. osmophila*; Km, *Kluyveromyces marxianus*; Kl, *K. lactis*; Sc, *S. cerevisiae*; Sk, *S. kudriavzevii*; Se, *S. eubayanus*; Wa, *Wickerhamomyces anomalus*; Wc, *W. ciferrii*. The evolutionary history was inferred by using the Maximum Likelihood method and JTT matrix-based model. The bootstrap consensus tree inferred from 100 replicates is taken to represent the evolutionary history of the taxa analyzed. The tree was generated using MEGA (Kumar *et al.* 2018).

.

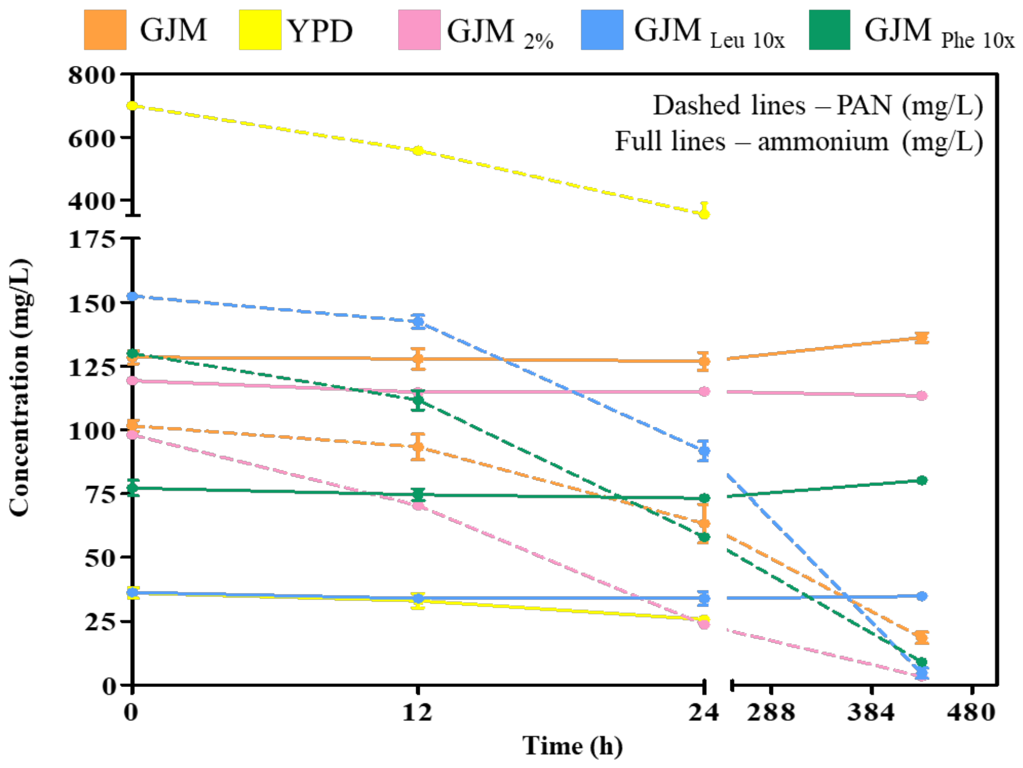

Supplementary Figure S6. Pattern of nitrogen consumption along fermentations of *H. guilliermondii* in YPD or in the different formulations of GJM utilized in this work ($GJM_{2\%}$, GJM_{Leu10x} and GJM_{Phe10x}). It is distinguished the consumption of primary amino nitrogen (which is estimated to be amino acids, based on the composition of the growth media) and of ammonium. Note the absence of ammonium consumption, while the levels of PAN are progressively reduced as the fermentations progress..

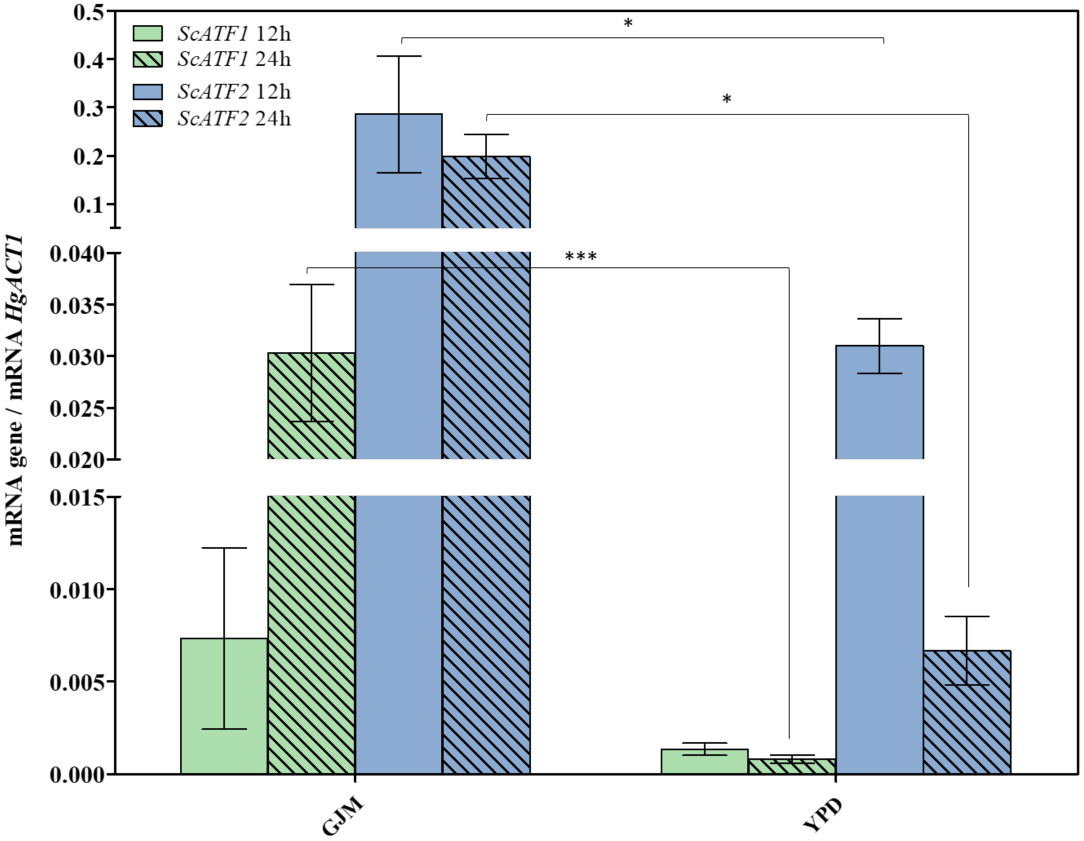

Supplementary Figure S7. Expression of *ScATF1* and ScATF2 along fermentations of *S. cerevisiae* UCD522 in GJM or in YPD. Fermenting cells cultivated were harvested 12 and 24h prior inoculation (the same time points at which the amount of higher alcohols and acetate esters were quantified) and total RNA extracted as detailed in materials and methods. Gene expression was quantified using real time RT-PCR. *ScACT1* was used as internal control and the gene expression represented corresponds to the ratio between the transcript level of each gene (corresponding to $2^{(-Ct)}$) and the transcript level obtained for *ScACT1* (corresponding to $2^{(-Ct)}$) in each sample. The results shown are the means of three independent experiments. Statistical significance between expression at the two time points was assessed using ANOVA, taking into account the different replicate assays performed. ***p<0.001, **p<0.01, *p<0.05.
